# Supplementary figures and images for: Sister centromere fusion during meiosis I depends on maintaining cohesins and destabilizing microtubule attachments
Source: PLoS Genet. 2019 May 31;15(5):e1008072. doi: 10.1371/journal.pgen.1008072 (PMC6581285; doi:10.1371/journal.pgen.1008072)

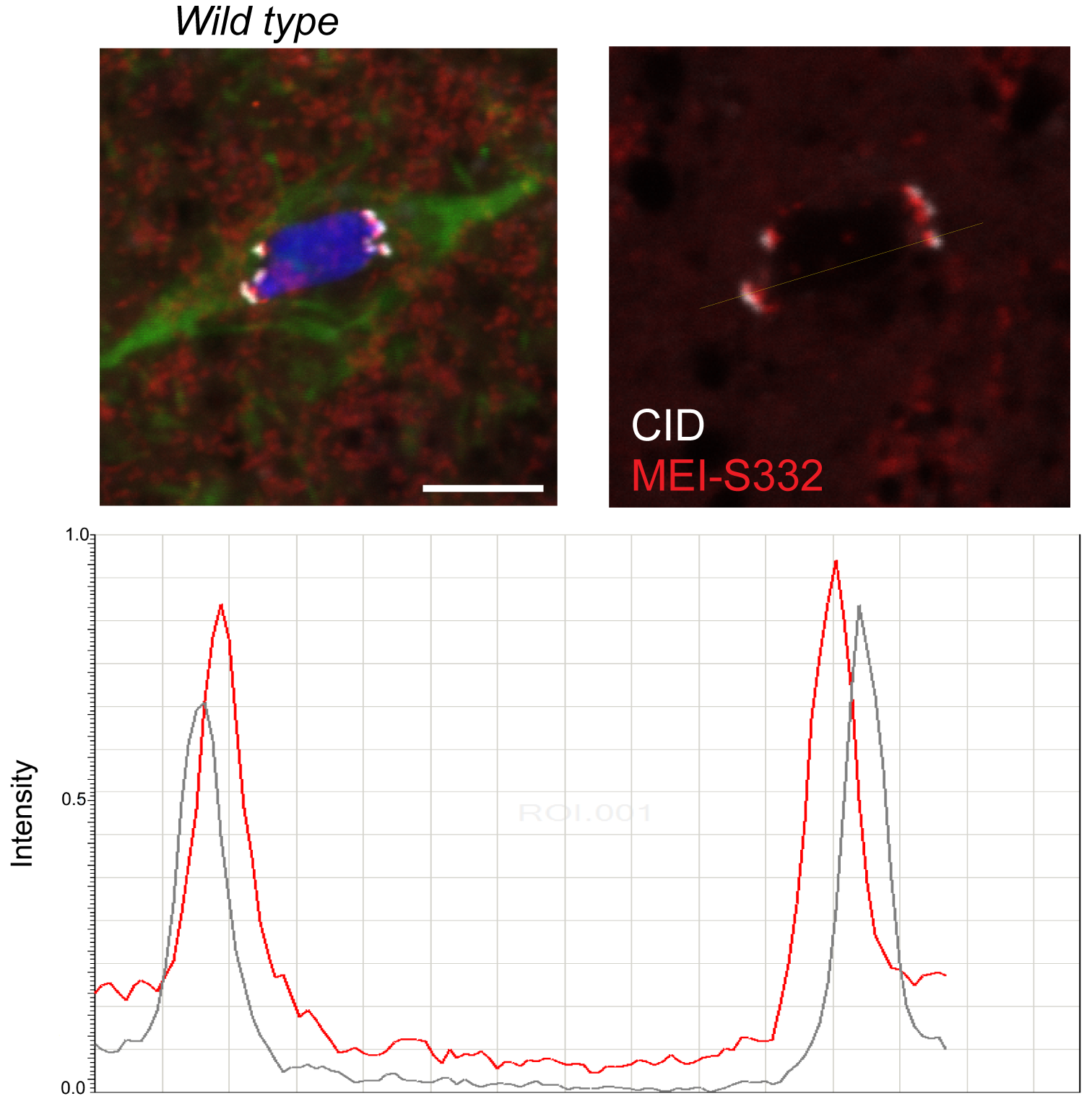

Supplement: S1 Fig — Representitive picture of wild type oocytes staining MEI-S332 (red) and CID (white) is shown and measured the intensity of flourensent. MEI-S332 localizes to both the pericentromeric and centromeric regions. Scale bar is 5 μm. (TIF) [file pgen.1008072.s001.tif]

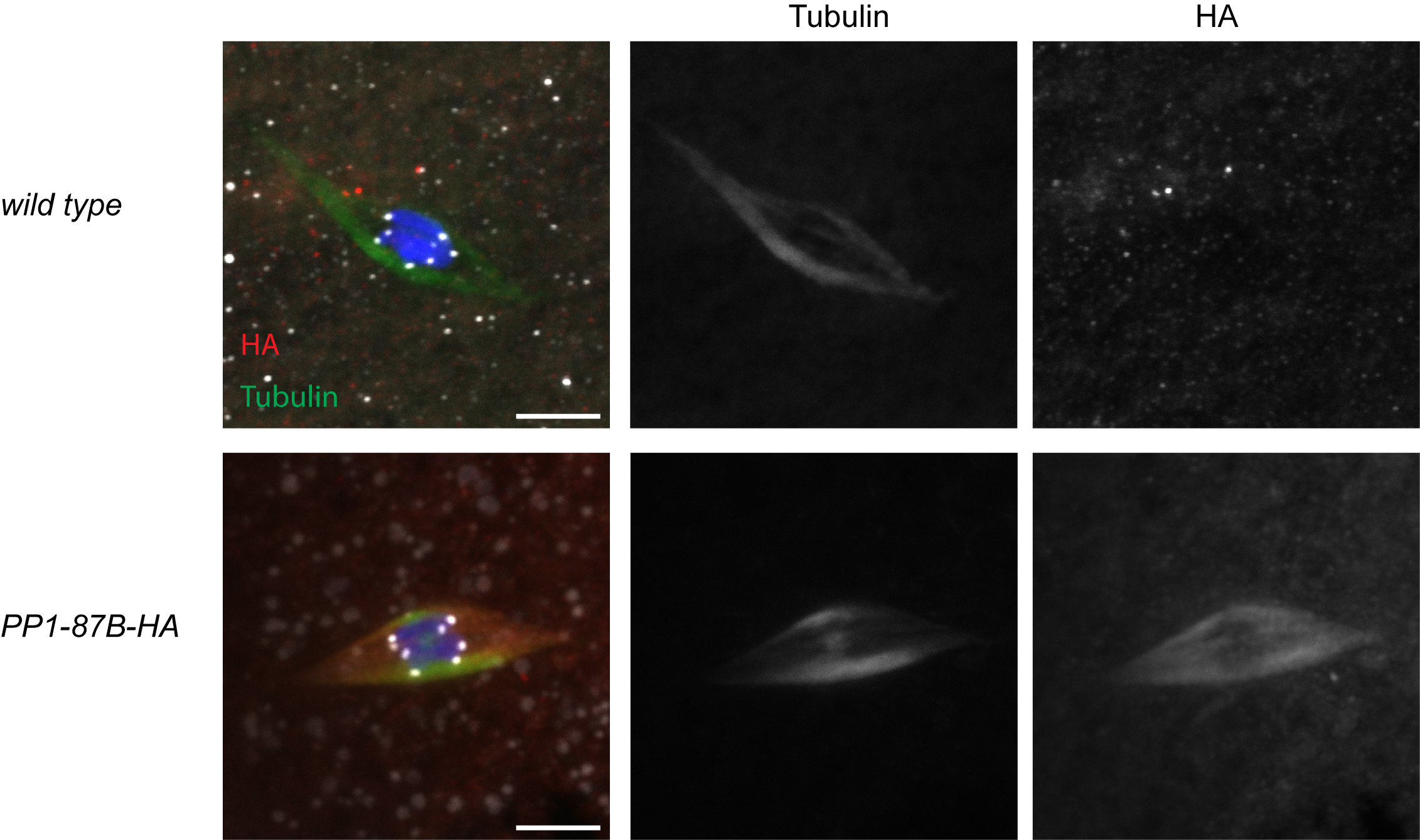

Supplement: S2 Fig — An epitope-tagged version of PP1-87B was expressed from a UASP transgene using mata4-GAL-VP16. HA-PP1-87B is in red, tubulin in green and DNA in blue and scale bars are 5 μm. (TIF) [file pgen.1008072.s002.tif]

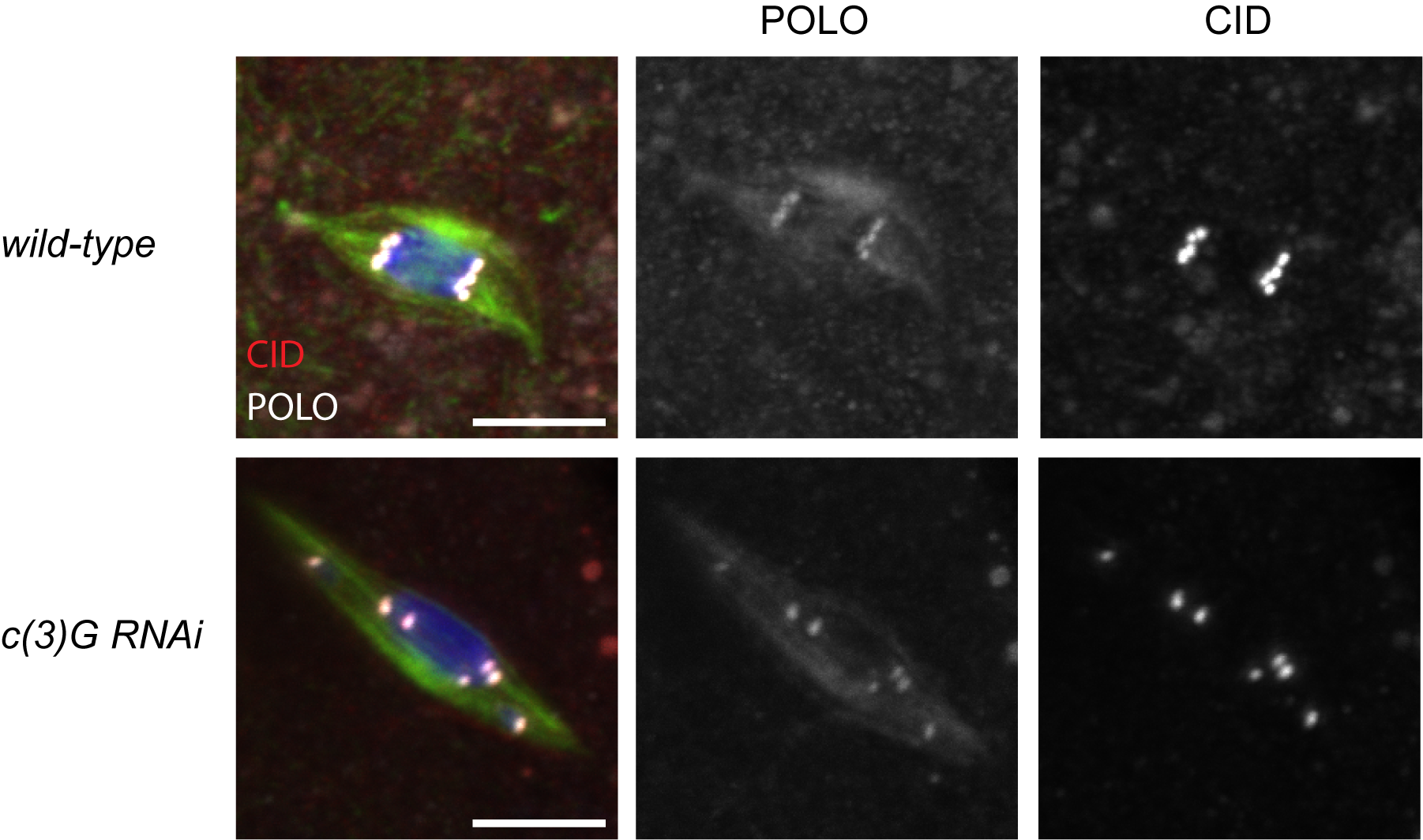

Supplement: S3 Fig — Wild-type, c(3)G RNAi, and Spc105R RNAi oocytes with DNA in blue, tubulin in green, Polo in red and CID in white. Single channels are shown in white. All images are maximum projections and scale bars are 5 μm. (TIF) [file pgen.1008072.s003.tif]

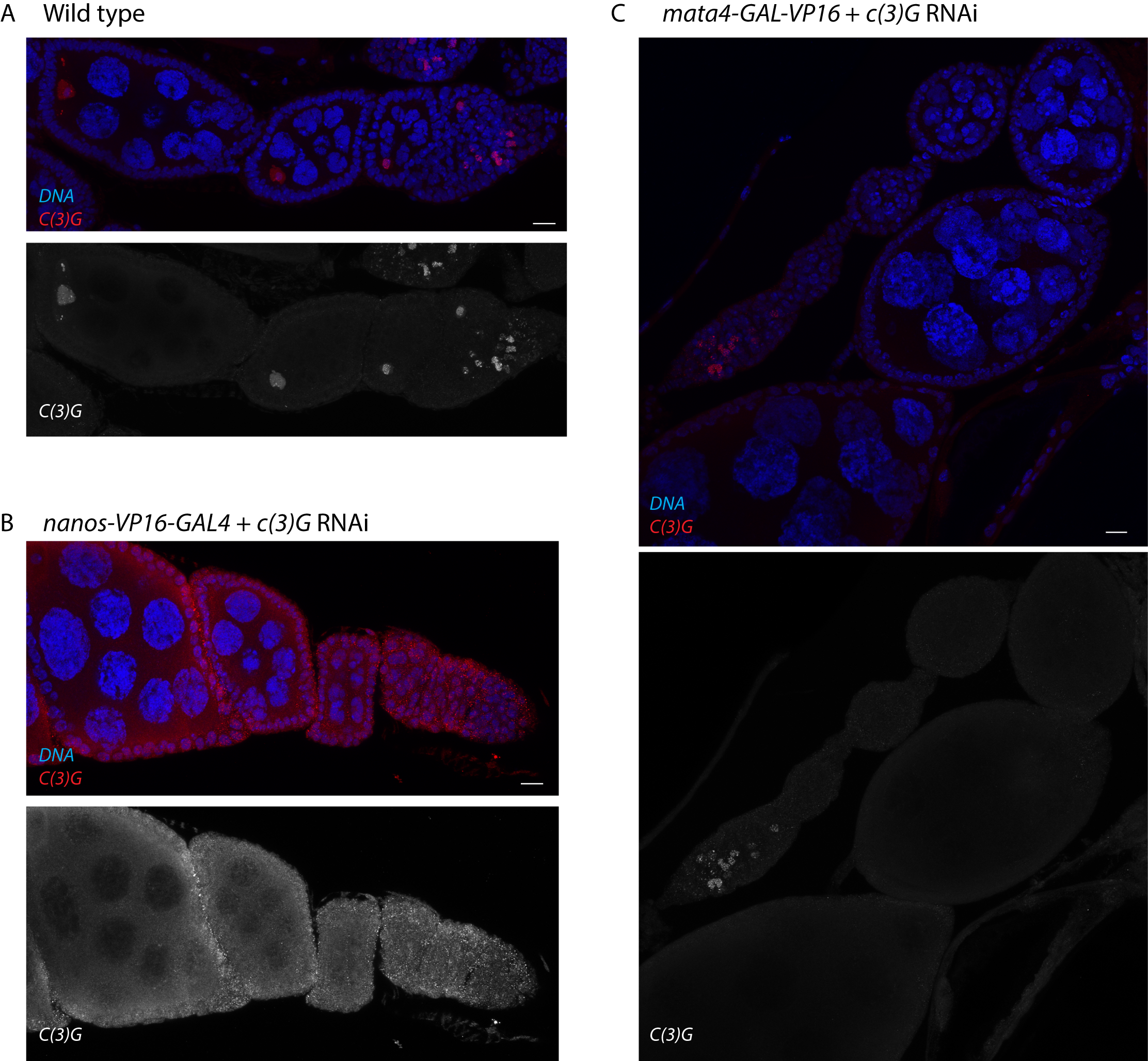

Supplement: S4 Fig — (A) C(3)G (red) forms thread-like structure in the germarium (early prophase), and retains them in oocytes of stages 2–5 of the vitellarium (late prophase). (B) When nanos-VP16-GAL4 expressed c(3)G shRNA in early prophase, C(3)G expression was abolished. (C) When mata4-GAL-VP16 expressed c(3)G shRNA in late prophase, C(3)G localization was present in germarium early pachytene, but absent in the stages 2–5 of the vitellarium. Scale bars are 10 μm. (TIF) [file pgen.1008072.s004.tif]

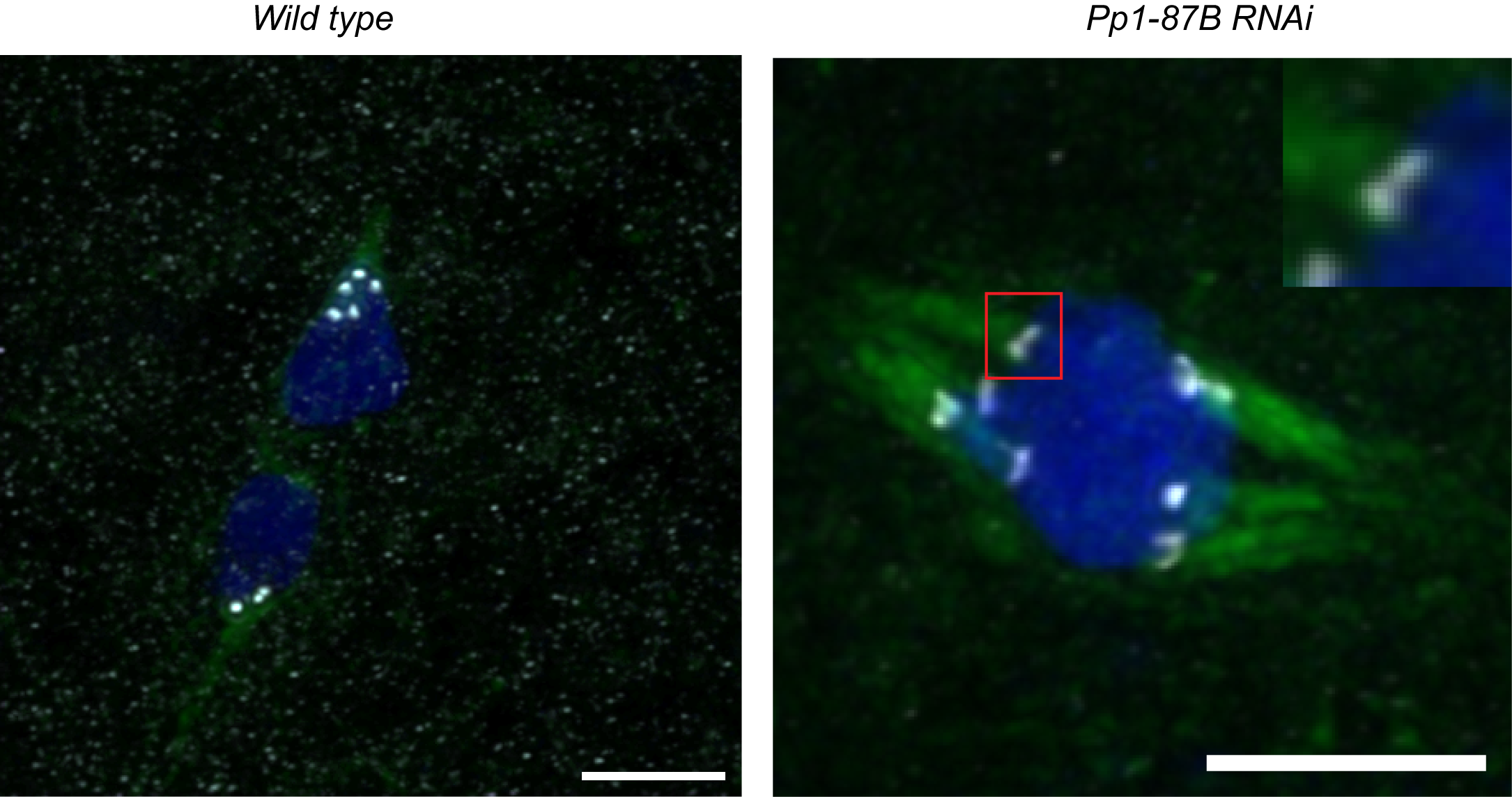

Supplement: S5 Fig — To observe whether the microtubule attachments in Pp1-87B RNAi oocytes are merotelic or syntelic in metaphase I, we used cold treatment to remove the unstable attachments. All females were cold treated for 2 hours before fixation. Presumably because depletion of PP1-87B stabilizes microtubule attachments, the Pp1-87B RNAi oocytes show a partial resistance to cold-treatment compared to wild-type. The images were taken and processed through deconvolution. All images are maximum projections and scale bars are 5 μm. (TIF) [file pgen.1008072.s005.tif]
